# Supplementary material for: Large-Scale Deployment and Establishment of Wolbachia Into the Aedes aegypti Population in Rio de Janeiro, Brazil
Source: Front Microbiol. 2021 Jul 29;12:711107. doi: 10.3389/fmicb.2021.711107 (PMC8356046; doi:10.3389/fmicb.2021.711107)
Supplement: Supplementary file 1 [file Data_Sheet_1.PDF]

Supplementary Information

Large-scale deployment and establishment of *Wolbachia* into the *Aedes aegypti* population in Rio de Janeiro, Brazil.

João Silveira Moledo Gesto[1,3+], Sofia Pinto[1,3+], Fernando Braga Stehling Dias[2,3], Julia Peixoto[3], Guilherme Costa[3], Simon Kutcher[4], Jacqui Montgomery[4], Benjamin R. Green[4], Katherine L. Anders[4], Peter A. Ryan[4], Cameron P. Simmons[4], Scott L. O'Neill[4], Luciano Andrade Moreira[1,3*]

[1] Grupo Mosquitos Vetores: Endossimbiontes e Interação Patógeno Vetor, Instituto René Rachou, Fiocruz Minas, Belo Horizonte, MG, Brazil.

[2] Gabinete da Presidência, Fiocruz, Rio de Janeiro, RJ, Brazil.

[3] World Mosquito Program, Fiocruz, Rio de Janeiro, RJ, Brazil.

[4] World Mosquito Program, Institute of Vector Borne Disease, Monash University, Clayton, 3800, Australia

* Correspondence: luciano.andrade@fiocruz.br

[+] These authors contributed equally to this work.

Supplementary Figure S1. Spatial distribution of adult-release sites. Approximate locations of release sites are depicted separately for each neighborhood of Ilha do Governador, and according to the deployment strategy: vehicle (blue dots) or backpack (red dots). A) RJ1.Bancários, B) RJ1.Cacuia, C) RJ1.Cocotá, D) RJ1.Freguesia, E) RJ1.Monero, F) RJ1.Pitangueiras, G) RJ1.Praia da Bandeira, H) RJ1.Ribeira, I) RJ1.Tauá, J) RJ1.Zumbi, K) RJ2.Cidade Universitária, L) RJ2.Galeão, M) RJ2.Jardim Carioca, N) RJ2.Jardim Guanabara, O) RJ2.Portuguesa. Urban perimeters enclosing release sites (black lines) filter out inhabited areas with low-abundance *Ae. aegypti*. Geotagged release site maps were generated using ArcGIS 10.4 (Esri, Redlands, CA, USA) and ©OpenStreetMap source data. Scale is represented at the bottom left corner.

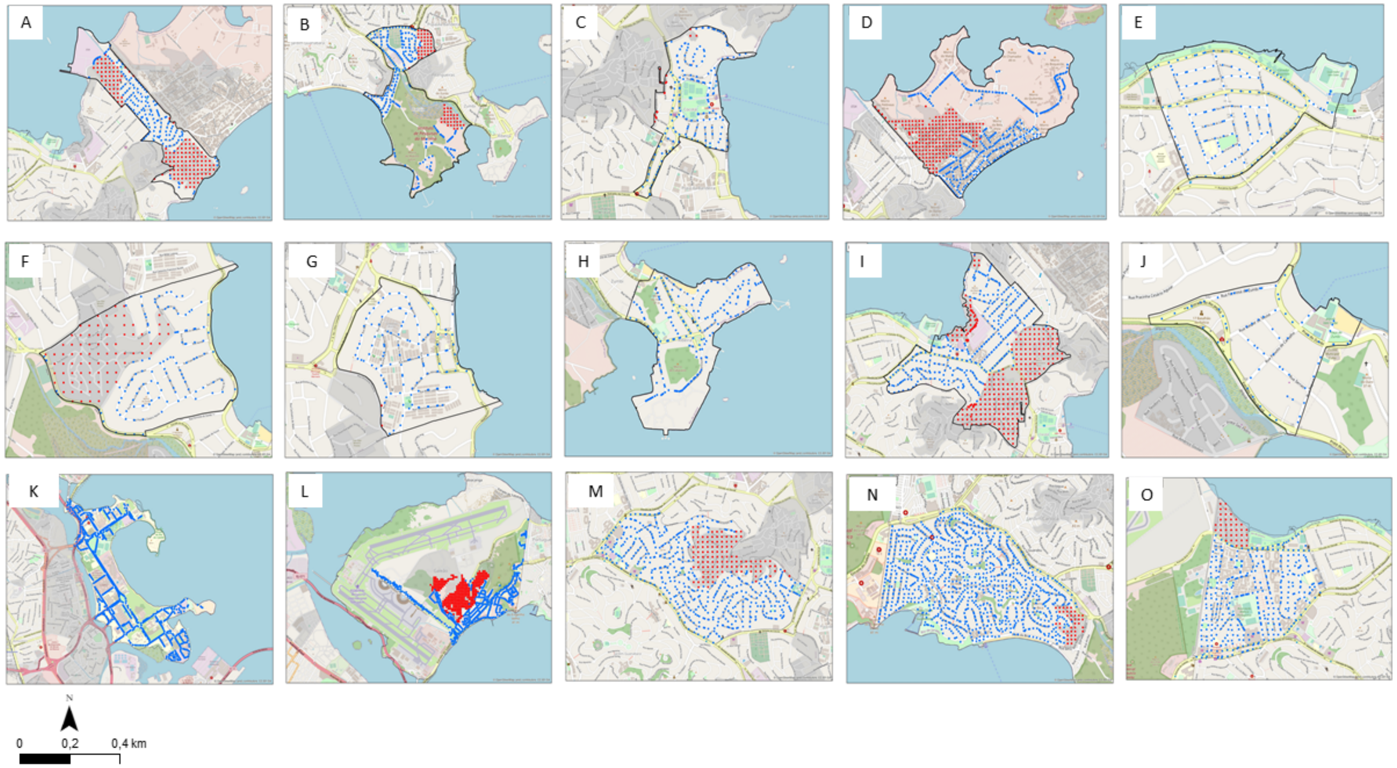

Supplementary Figure S2. Spatial distribution of monitoring sites. Geotagged locations of BG-sentinel traps set in household partners are depicted separately for each neighborhood of Ilha do Governador, according to deployment strategy used: vehicle (black dots) or backpack (purple dots). A) RJ1.Bancários, B) RJ1.Cacuia, C) RJ1.Cocotá, D) RJ1.Freguesia, E) RJ1.Monero, F) RJ1.Pitangueiras, G) RJ1.Praia da Bandeira, H) RJ1.Ribeira, I) RJ1.Tauá, J) RJ1.Zumbi, K) RJ2.Cidade Universitária, L) RJ2.Galeão, M) RJ2.Jardim Carioca, N) RJ2.Jardim Guanabara, O) RJ2.Portuguesa. Urban perimeters (black lines) filter out non-inhabited areas with low-abundance *Ae. aegypti*. Trapped specimens were assessed for *Wolbachia* frequency status. Maps were created with ArcGIS 10.4 (Esri, Redlands, CA, USA) and ©OpenStreetMap source data. Scale is represented at the bottom left corner.

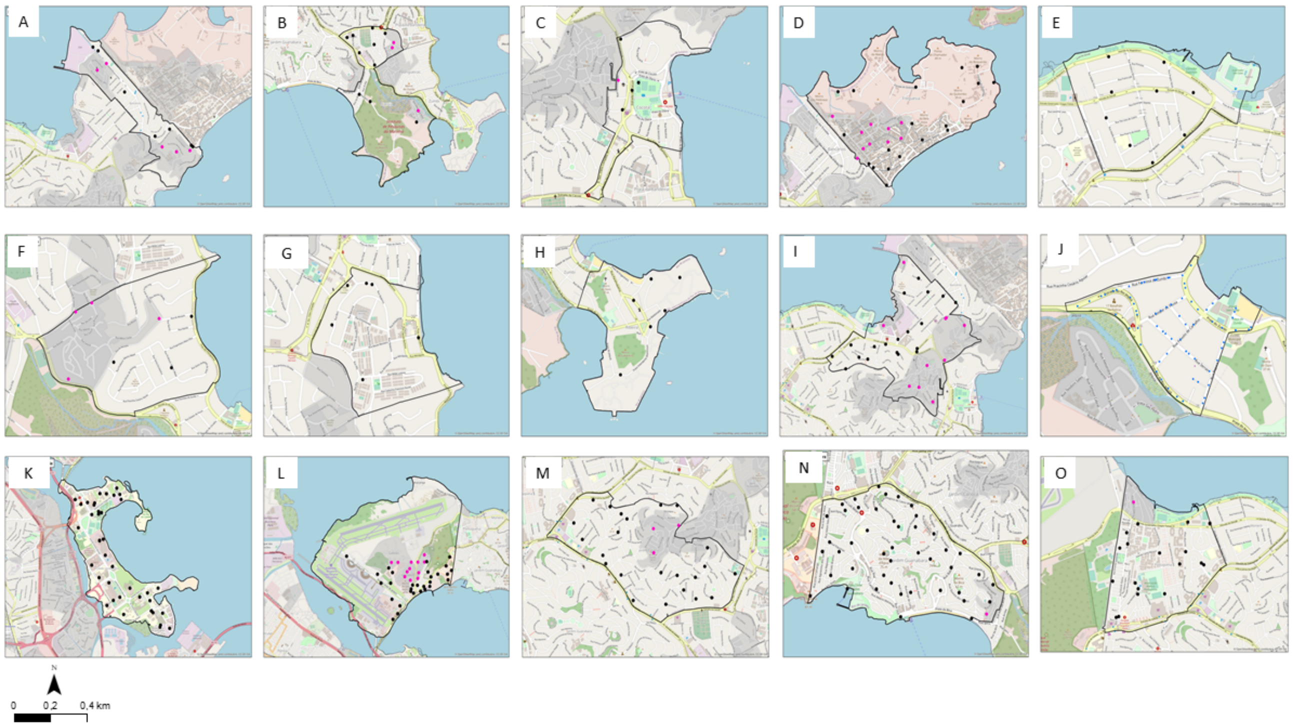

Supplementary Table S1. Adult-release schedules and dosages across intervention areas. Schedules and numbers of released mosquitoes per intervention area, RJ1 or RJ2, and deployment strategy, vehicle (V) or backpack-assisted (B).

Area	Begin	End	Weeks	Events[a]	Sites/ event[b] (± SD)	Released/ event[c] (± SD)	Released/ event/ site[d] (± SD)	Released/ event/ area[e] (± SD)	Released (Total)
**1st Round**									
RJ1.B	29/08/2018	01/02/2018	20	10	871 (± 0)	100,601 (± 42,932)	118 (± 45)	48,836 (± 20,841)	1,006,012
RJ1.V	29/08/2017	02/01/2018	20	14	1,668 (± 642)	228,159 (± 93,244)	137 (± 14)	36,623 (± 14,967)	3,194,229
RJ2.B	21/11/2017	16/01/2018	9	8	763 (± 0)	77,462 (± 44,269)	101 (± 58)	56,132 (± 32,079)	619,699
RJ2.V	23/11/2017	25/01/2018	10	9	4,657 (± 1,348)	419,655 (± 329,660)	91 (± 60)	27,215 (± 21,379)	3,776,895
**2nd Round**									
RJ1.B	13/03/2018	05/06/2018	13	12	967 (± 120)	83,265 (± 35,945)	93 (± 31)	40,420 (± 17,449)	999,184
RJ1.V	23/02/2018	14/06/2018	17	15	2,387 (± 554)	233,504 (± 49,424)	100 (± 17)	37,481 (± 7,933)	3,502,563
RJ2.B	15/05/2018	28/08/2018	16	16	748 (± 12)	83,574 (± 19,516)	112 (± 24)	60,561 (± 14,142)	1,337,184
RJ2.V	08/05/2018	31/08/2018	18	19	4,097 (± 1,353)	453,681 (± 173,901)	112 (± 19)	29,422 (± 11,278)	8,619,954
**3rd Round**									
RJ1.B	13/03/2019	04/07/2019	17	12	134 (± 0)	14,483 (± 4,800)	108 (± 36)	7,031 (± 2,330)	173,798
RJ1.V	01/03/2019	12/06/2019	16	16	291 (± 89)	26,472 (± 10,137)	91 (± 20)	4,249 (± 1,627)	423,558
RJ2.B	13/06/2019	19/09/2019	28	22	71 (± 58)	5,983 (± 4,681)	102 (± 30)	6,729 (± 910)	204,279
RJ2.V	30/04/2019	13/08/2019	16	16	2,190 (± 533)	215,596 (± 71,851)	97 (± 21)	13,982 (± 4,660)	3,449,540

[a] Number of events (weeks) that release intervention occurred.
[b] Number of release sites per event.
[c] Number of mosquitoes released per event.
[d] Number of mosquitoes released per event per site (i.e. number of mosquitoes in each release tube).
[e] Number of mosquitoes released per event per intervention area (km^2).

Supplementary Table S2. BG-Sentinel traps allocation. Number of BG-Sentinel traps allocated to each neighborhood section of Ilha do Governador, from the beginning until the end of field monitoring. Revision of schedules and trap numbers was necessary to manage a long-term monitoring activity, and obtain the best possible spatial resolution.

**Intervention section**	**Number of BG-traps**			**Monitoring schedule**	
	Max.	Min.	Mean (± SD)	Start	End
RJ1.V.Bancários	7	3	5.9 ± 1.1	10/2017	03/2020
RJ1.V.Cacuia	10	2	6.6 ± 2.9	10/2017	03/2020
RJ1.V.Cocotá	4	1	2.9 ± 0.9	10/2017	03/2020
RJ1.V.Freguesia	26	1	13.1 ± 5	10/2017	03/2020
RJ1.V.Monero	9	5	6.7 ± 2	10/2017	03/2020
RJ1.V.Pitangueiras	3	2	2.4 ± 0.5	10/2017	03/2020
RJ1.V.Praia da Bandeira	6	2	4.1 ± 1.6	10/2017	03/2020
RJ1.V.Ribeira	7	2	4.2 ± 1.5	10/2017	03/2020
RJ1.V.Tauá	18	8	12 ± 4.4	10/2017	03/2020
RJ1.V.Zumbi	3	1	1.8 ± 1	10/2017	03/2020
RJ1.B	35	1	29.2 ± 8.1	10/2017	03/2020
RJ2.V.Cidade Universitária	41	42	41.6 ± 0.5	12/2017	03/2020
RJ2.V.Galeão	31	1	29.3 ± 3	12/2017	03/2020
RJ2.V.Jardim Carioca	23	1	21.4 ± 3.5	12/2017	03/2020
RJ2.V.Jardim Guanabara	46	3	39 ± 12.2	12/2017	03/2020
RJ2.V.Portuguesa	18	2	17.5 ± 2.6	12/2017	03/2020
RJ2.B	26	15	23.1 ± 2.2	12/2017	03/2020
Tubiacanga	15	3	5.4 ± 4.6	10/2017	03/2020

Supplementary Table S3. Nucleotide sequences of primers and probes. List of primers and probes used for the molecular diagnostics of *Wolbachia* by qPCR and LAMP, and for *kdr* genotyping.

PRIMER	NUCLEOTIDE SEQUENCE (5' → 3')
Ae. aegypti RPS17 – qPCR	
RPS17S Forward	TCCGTGGTATCTCCATCAAGCT
RPS17S Reverse	CACTTCCGGCACGTAGTTGTC
RPS17S Probe	**HEX**/CAGGAGGAG/**ZEN**/GAACGTGAGCGCAG/**3lABkFQ**
Ae. aegypti kdr screening – qPCR	
1016 Forward	CGTGCTAACCGACAAATTGTTTCC
1016 Reverse	GACAAAAGCAAGGCTAAGAAAAGGT
1016 Probe Val+	**VIC**/CCGCACAGATACTTA/**NFQ**
1016 Probe Ile^{kdr}	**FAM**/CCCGCACAGGTACTTA/**NFQ**
1534 Forward	CGAGACCAACATCTACATGTACCT
1534 Reverse	GATGATGACACCGATGAACAGATTC
1534 Probe Phe+	**FAM**/ACGACCCGAAGATGA/**NFQ**
1534 probe Cys^{kdr}	**VIC**/AACGACCCGCAGATGA/**NFQ**
Wolbachia – qPCR	
WSPTM2 Forward	CATTGGTGTTGGTGTTGGTG
WSPTM2 Reverse	ACACCAGCTTTTACTTGACCAG
WSPTM2 Probe	**FAM**/TCCTTTGGA/**ZEN**/ACCCGCTGTGAATGA/**3lAbRQSp**
Wolbachia – LAMP	
FIP	TGTATGCGCCTGCATCAGCTTCGGTTCTTATGGTGCTAA
BIP	GCAGAAGCTGGAGTAGCGTTGTGTCATGCCACTTAGATGG
F3	TGATGTAACTCCAGAAGTCA
B3	CTTATTGGACCAACAGGATCG
LpF	AGCCTGTCCGGTTGAATT
LpB	CAGTCTTGTTATCCCAGTGAGT
